# Novel Heteroatom-Doped Fe/N/C Electrocatalysts With Superior Activities for Oxygen Reduction Reaction in Both Acid and Alkaline Solutions

**DOI:** 10.3389/fchem.2020.00078

**Published:** 2020-02-18

**Authors:** Muhammad Rauf, Jingwen Wang, Waheed Iqbal, Mazhar Abbas, Sayed Ali Khan, Qudrat Ullah Khan, Xiangzhong Ren, Peixin Zhang, Yongliang Li

**Affiliations:** ^1^College of Chemistry and Environmental Engineering, Shenzhen University, Shenzhen, China; ^2^Environmental Science and Engineering Research Center, Harbin Institute of Technology, Shenzhen, China; ^3^Shenzhen Key Laboratory of Micro-Scale Optical Information Technology, Nanophotonics Research Center, Shenzhen University, Shenzhen, China; ^4^College of Physics and Optoelectronic Engineering, Shenzhen University, Shenzhen, China; ^5^Guangdong Flexible Wearable Energy and Tools Engineering Technology Research Centre, Shenzhen University, Shenzhen, China

**Keywords:** heteroatoms doped catalysts, fuel cells, oxygen reduction reaction, acid solution, active sites

## Abstract

The exploration of noble metal-free catalysts with efficient electrochemical performance toward oxygen reduction reaction in the acid electrolyte is very important for the development of fuel cells technology. Novel pyrolyzed heteroatom-doped Fe/N/C catalysts have been regarded as the most efficient electrocatalytic materials for ORR due to their tunable electronic structure, and distinctive chemical and physical properties. Herein, nitrogen- and sulfur-doped (Fe/N/C and Fe/N/C-S) electrocatalysts were synthesized using ferric chloride hexahydrate as the Fe precursor, N-rich polymer as N precursor, and Ketjen Black EC-600 (KJ600) as the carbon supports. Among these electrocatalysts, the as prepared S and N-doped Fe/N/C-S reveals the paramount ORR activity with a positive half-wave potential value (*E*_1/2_) 0.82 at 0.80 V vs. RHE in 0.1 mol/L H_2_SO_4_ solution, which is comparable to the commercial Pt/C (Pt 20 wt%) electrocatalyst. The mass activity of the Fe/N/C-S catalyst can reach 45% (12.7 A g^−1^ at 0.8 V) and 70% (5.3 A g^−1^ at 0.95 V) of the Pt/C electrocatalyst in acidic and alkaline solutions. As result, ORR activity of PGM-free electrocatalysts measured by the rotating-ring disk electrode method increases in the following order: Fe/N/C<Fe/N/C-S, in both basic and acidic medium. This scientific work offers a facile approach to design and synthesizes efficient heteroatom-doped catalytic materials for electrochemical reactions in energy devices.

## Introduction

The electrochemical oxygen reduction reaction is the most significant process at cathode in the polymer electrolyte membrane fuel cell (PEMFC) and anion-exchange membrane alkaline fuel cell (AEMFC). The high-cost platinum and other noble metals based electrocatalysts are still commonly used for ORR because of their unique characteristics, such as low over-potential and high current density (Marković et al., 2001; Chung et al., 2017; Gottesfeld et al., 2018). It is known that more electrocatalyst loading is needed (~80% of the Pt) to ameliorate the sluggish oxygen reduction process at cathode in fuel cells (Wu and Zelenay, 2013). However, the large-scale fuel cell applications are confined owing to high-cost, the fact they are easily poisoning to CO and methanol, have limited supply and long-term stability, low electrocatalytic selectivity, and also due to the sluggish reaction kinetics of noble metal-doped (Pt, Pd, Ru, etc.) catalysts for ORR (Rauf et al., 2018; Wang Y. et al., 2018). To tackle these challenges, cost-effective noble metals-free catalysts are an alternative and effective approach to develop fuel cell technology (Lim et al., 2009). Therefore, it is urgent to develop noble metals-free electrocatalytic nanomaterials with superior activity and durability, in order to commercialize fuel cell devices on a large-scale.

In the last decade, enormous efforts have been made for developing high-performance and cost-effective noble metal-free catalysts for ORR (Dai et al., 2015; Chung et al., 2017; Gewirth et al., 2018; Li et al., 2018; Xue et al., 2018). These electrocatalysts were based on various kinds of heteroatoms, transition metals, nitrogen and carbon precursors, such as carbon-supported nitrogen and transition metals-doped catalytic materials (Bezerra et al., 2008; Jaouen et al., 2011; Higgins and Chen, 2013; Rauf et al., 2016), dual heteroatom-doped carbon nanotubes or graphene (Yao et al., 2012; Nyoni et al., 2015; Rauf et al., 2017), metals-N_4_ macrocycles (Jasinski, 1964; Seo et al., 2014), heteroatoms doped carbon nanomaterials (Zhang et al., 2016; Liu et al., 2019; Wu et al., 2019), etc., which showed high catalytic activity, stability, and high tolerance to small alcohol molecules or CO poisoning (Qu et al., 2010). Among them, carbon-supported nanomaterial with heteroatoms doping (e.g., Co, Fe, N, S, P, and halogens) were widely developed, and they are promising non-precious electrocatalysts to substitute Pt group metal (PGM)-free electrocatalysts for ORR (Gong et al., 2009; Yang et al., 2012; Yao et al., 2012; Zhang and Dai, 2012; Jeon et al., 2013; Chen et al., 2017; You et al., 2018). Sulfur and halogens (F and I) had been introduced as active additives in Fe/N/C or Co/N/C electrocatalysts to improve the electrocatalytic performance (Chen et al., 2015; Nyoni et al., 2015; Wang Y.-C. et al., 2018; Zheng et al., 2019). The dual-doping of non-precious metals (Fe, Co, etc.) and heteroatoms could be attributed to the accessibility of reactants for ORR and an increase in the charge transfer rate at the electrode/electrolyte interface (Li et al., 2015). It is found that the different atomic size and electronegativity of heteroatoms enhanced the ORR current density by changing the charge distribution of contiguous carbon atoms, and created the active sites center in electrocatalysts (Wang et al., 2011; Choi et al., 2012). The heteroatom-doped Fe/N/C electrocatalysts had shown higher ORR performance in basic solution as compared to the acid solution, due to different reaction mechanisms under different pH values of electrolyte solution (Rauf et al., 2016; Gewirth et al., 2018). It is reported that the presence of sulfur-based species can enhance the ORR performance of Fe/N/C catalysts by decreasing the generation of intermediate H_2_O_2_. However, there are a number of reports which concentrate on the heteroatom-doped Fe/N/C electrocatalysts, and most of them are investigating the ORR performance in alkaline solutions (Hoque et al., 2018; You et al., 2018; Wu et al., 2019; Zheng et al., 2019). Therefore, it is necessary to develop heteroatom-doped catalysts with high electrochemical performance in acidic medium, which so far is more practical for fuel cells. Moreover, another challenge is the uniform doping of heteroatoms in a facile and controllable way in the electrocatalysts.

In this report, we synthesized heteroatom-doped Fe/N/C electrocatalysts with the porous structure for ORR, to especially improve the activity in acidic electrolyte. Among them, the Fe/N/C-S catalyst with uniform potential catalytic active sites, high surface areas (830 m^2^g^−1^) and porosity demonstrated best electrochemical performance. The half-wave potential (*E*_1/2_) reaches to 944 mV (vs. RHE), that is 34 mV more positive (910 mV vs. RHE) than a commercial Pt/C electrocatalyst and high limiting current density of 5.5 mA cm^−2^ at 0.43 V in 0.1 M sodium hydroxide solution. While in 0.1 M H_2_SO_4_ electrolyte solution, *E*_1/2_ potential of the Fe/N/C-S electrocatalyst reaches 820 mV (vs. RHE), which is only 64 mV less than Pt/C electrocatalyst. From the surface morphology analysis, the Sulfur-doped Fe/N/C electrocatalyst looks mesoporous and the Fe particles are preserved through graphitic layers. This electrocatalyst showed high ORR performance, low H_2_O_2_% yield, and followed the four-electron path selectivity due to uniform doping of heteroatoms N, S and Fe metal.

## Experimental Section

### Reagents and Materials

Melamine (99%), Terethalaldehyde (98%), and dimethyl sulphoxide (99%) were received from Aladdin reagents (Shanghai) Co., LTD. Ferric chloride hexahydrate (FeCl_3_.6H_2_O), 99.0%), hydrochloric acid (36%), ethanol (99.7+%), Superpur Sulfuric acid (96.0%), Calcium Hydride (CaH_2_) and sodium hydroxide (98.0%) were obtained from China Chemical Reagent Corporation Aladin and Macklin. The commercial Pt/C (Pt 20 wt%) and Nafion (5%) were purchased from Alfa Aesar. The carbon black (Ketjenblack EC600J) was bought from Akzo Nobel, Japan. The deionized water (18.2 MΩ) was used during all experimental work.

### Synthesis of Fe-N/C

**Figure 1:**
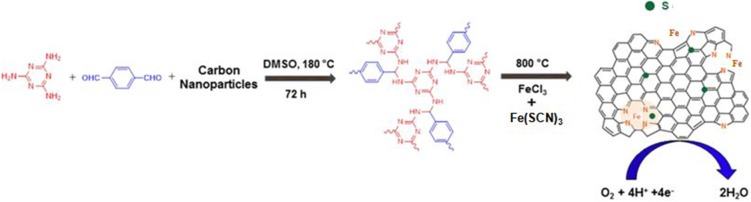
The summary of synthesis of dual-doped electrocatalysts.

The N-doped Fe-N/C electrocatalyst was prepared as in our previous report (Rauf et al., 2016). In brief, the N-rich polymer as nitrogen source was synthesized through the reaction between melamine (5.0 mmol) and terephthalaldehyde (7.5 mmol) with DMSO solvent (18 mL) in 25 ml Teflon lined autoclave reactor at 180°C for 72 h. The formed N-rich polymer was *in-situ* coated on KJ600 carbon black (ca. 0.5 g). The synthesized N-doped carbon material (1 g) was mixed with ferric chloride (3 mmol) through C_2_H_5_OH solvent by magnetic stirring. The ethanol was evaporated through a vacuum rotary evaporator machine and further dried at 50°C for 10 h. The resulting dried carbon material was pyrolyzed at high temperature 800°C under inert atmosphere (Ar gas) for 1 h with the heating rate of 5°C per min (HT1). The pyrolyzed powder was dispersed in 0.1 M super sulphuric acid by stirring at 80°C for 8 h (HT1-AL). The unstable and inactive impurities were removed by centrifugation and washing with pure H_2_O several times. The washed product was dried at 85°C for a whole night and then again pyrolyzed (HT2) at 800°C for another 3 h with a heating rate of 5°C min^−1^ to get the final Fe-N/C electrocatalyst (Figure 1).

### Preparation of S-Doped Fe-N/C

To synthesize S-doped Fe-N/C, firstly, we prepared fresh Fe(SCN)_3_ solution by mixing 1 M FeCl_3_ with 1 M KSCN in 1:3 ratio. Secondly, N-doped carbon (N/C: 0.5 g) was mixed with freshly prepared Fe(SCN)_3_ solution by magnetic stirring and dried at 80°C overnight. The final S-doped Fe-N/C catalyst was obtained after performing a heat treatment and acid leaching process as like to the Fe/N/C electrocatalyst.

### Physical Characterizations

The surface morphologies and microstructure of electrocatalysts were analyzed by a field emission scanning electron microscope (JEOL JSM-7800F) and a high-resolution transmission electron microscopy (JEM-2100 and X-Max 80), respectively. The X-ray diffraction testing was done by D8 Advance with Cu Kα radiations. The surface chemical composition and active species of the electrocatalytic materials were examined by X-ray photoelectron spectroscopy (XPS) with an Ultra DLD using a monochromic Al X-ray source. The Ar adsorption/desorption isotherms were obtained by a Micromeritics (ASAP 2020) device. The pore size distribution and specific surface areas data were calculated by using the Brunauer-Emmett-Teller theory. The Raman spectra were recorded by using a Renishaw/Invia Reflex spectrometer coupled with a 633 nm laser.

### Electrochemical Experiment Section

The electrochemical testing of electrocatalysts was evaluated by using a CHI-760D bipotentiostat (China) and three electrodes connected to an electrolytic cell at a constant temperature 30°C. The saturated calomel electrode (SCE) and mercury/mercury oxide (Hg/HgO) were used as reference electrodes. A thin graphite strip was used as a counter electrode. A glassy carbon (diameter = 5.61) surrounded by a Pt-metal ring was used as a substrate for the preparation of working electrode.

For the preparation of working electrode, the required amount of catalyst (6–10 mg) was ultrasonically dissolved in 1 mL solution (0.5 mL C_2_H_5_OH, 50 μL 5% Nafion and 0.45 mL H_2_O). A total 25 μL of the catalyst solution was dropped on the glassy carbon disk electrode after polishing by 0.3 μm Al_2_O_3_, and dried at room temperature. For comparison purposes, commercial Pt/C (Pt 20 wt%) electrocatalyst ink was synthesized with a similar method, by dissolving 1 mg in 1 mL C_2_H_5_OH solvent. The cyclic voltamograms were measured by potential cycling between 0.2 and 1.2 V vs. RHE with 900 rpm at 10 mV s^−1^ scan rate. The electrolytic solutions were saturated with O_2_ gas for 30 min before the measurements and ohmic drop value (iR drop) was compensated. The CV curves also recorded in nitrogen gas saturated electrolyte solution under similar conditions. The current recorded in the O_2_-saturated electrolyte was revised by the background capacitive current to obtain the ORR current. The mass activity of the electrocatalyst was calculated by dividing the kinetic current (*J*_*k*_) to the catalyst loading. The Koutecky-Levich's (K-L) equation (Equation 1) applied to calculate the kinetic current.

(1) $$\begin{array}{l} \frac{1}{j}=\frac{1}{j_{k}}+\frac{1}{j_{L}} \end{array}$$

The H_2_O_2_ yield was determined through Pt ring at 1.3 V vs. RHE, and the H_2_O_2_ percentage was determined by Equation 2.

(2) $$\begin{array}{l} {\text{H}}_{2}{\text{O}}_{2}\left(\;\%\;\right)=200*\frac{{\text{I}}_{\text{ring}}/\text{N}}{{\text{I}}_{\text{disk}}+{\text{I}}_{\text{ring}}/\text{N}} \end{array}$$

The collection efficiency of RRDE was experimentally measured to be 0.386 in 5 mM K_4_Fe(CN)_6_ and 1 M Sr(NO_3_)_2_ solution. The selectivity of the ORR process can be determined through H_2_O_2_ percentage by the Equation 3.

(3) $$\begin{array}{l} \text{ne}=4*\frac{{\text{I}}_{\text{disk}}}{{\text{I}}_{\text{disk}}+\frac{{\text{I}}_{\text{ring}}}{\text{N}}} \end{array}$$

## Results and Discussion

**Figure 2:**
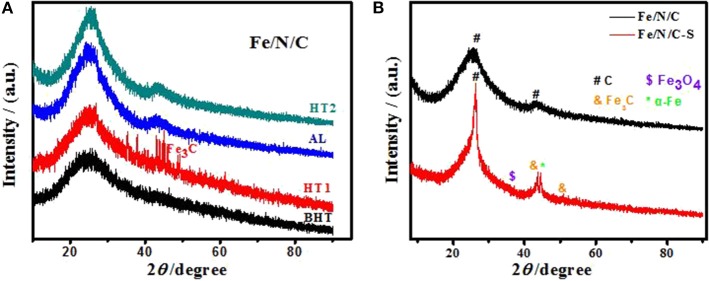
XRD patterns of **(A)** the Fe/N/C electrocatalyst at different preparation stages, **(B)** the Fe/N/C and Fe/N/C-S electrocatalysts after HT2.

The structural properties of electrocatalysts were analyzed through X-ray diffraction. The XRD pattern of electrocatalyst showed characteristic reflections of crystalline iron carbide (Fe_3_C) and other metal impurities after first heat treatment (HT1) in Figure 2A. Metal impurities formed after HT1 were washed out by an acid leaching process (HT1-AL). The XRD patterns showed broad and prominent peaks centering at the 2θ angles of 26.2° and 43°, which were assigned to the graphitic carbon framework of (002) and (100) planes, respectively. These strong peaks were formed due to the heteroatoms doping in electrocatalysts, and showed that the graphitic layers increased after the pyrolysis at high temperature. There is no prominent peak of Fe metal in the XRD pattern of the Fe/N/C electrocatalyst as compared to the Fe/N/C-S sample (Figure 2B). However, there are some other confirmed characteristic peaks that correspond to the Fe_3_C with PDF No. 23-0298 and Fe_3_O_4_ with PDF No. 03-0863 in the Fe/N/C-S electrocatalyst. The XRD results proved that the amount of iron is quite higher after the acid leaching and second heat treatment, which indicates that Fe nanoparticles were protected due to N-doped carbon nanoshells in the S-doped electrocatalyst.

**Figure 3:**
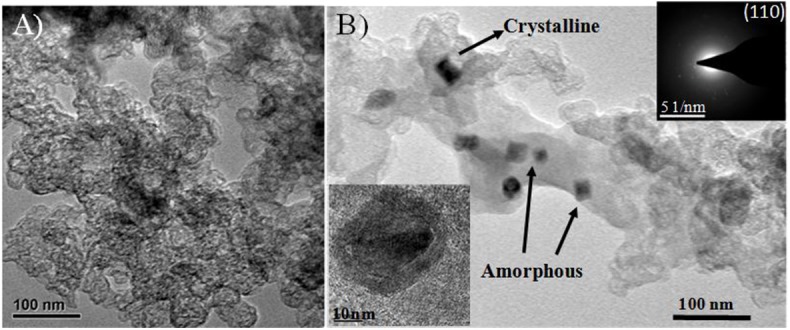
Morphology characterization of dual heteroatom-doped electrocatalysts: **(A)** TEM image of the Fe/N/C, **(B)** TEM images of the Fe/N/C-S catalyst. The insets are the HR-TEM image of covered Fe particle and corresponding SAED pattern.

The transmission electron microscope images of heteroatom-doped electrocatalysts are shown in Figure 3. The surface morphology of the N-doped catalyst Fe/N/C seems like the agglomeration of carbon nanoparticles, as can be seen in Figure 3A where the size of the nanoparticles is ~30 nm. The Fe/N/C-S catalyst possessed a network-like mesoporous structure, which is clearly seen in the TEM image of the electrocatalyst (Figure 3B). In the Fe/N/C-S catalyst, N-rich polymer-coated carbon black precursor engrossed with Fe and S sources and sustained well-defined and smooth outer surfaces. TEM results are indicating that the Fe and S doping were highly dispersed in the pores of the carbon materials. For the Fe/N/C-S catalyst, iron particles identified in amorphous and crystalline form on the surface of the electrocatalyst. There are some Fe particles covered by carbon nanoshells/graphitic layers which even survived after the acid leaching and second heat treatment process. Furthermore, covered Fe particles were confirmed by HR-TEM analysis (inset image of Figure 3B). The SAED patterns (inset of Figure 3B) justify the crystalline structure of covered particle α-Fe (110) in Fe/N/C-S electrocatalyst. The heteroatoms Fe, N, and S, might have been well-immersed and integrated into the carbon framework in Fe/N/C-S, rather than agglomerating on the surface like the Fe/N/C catalyst, which may result in improved electrochemical performance.

**Figure 4:**
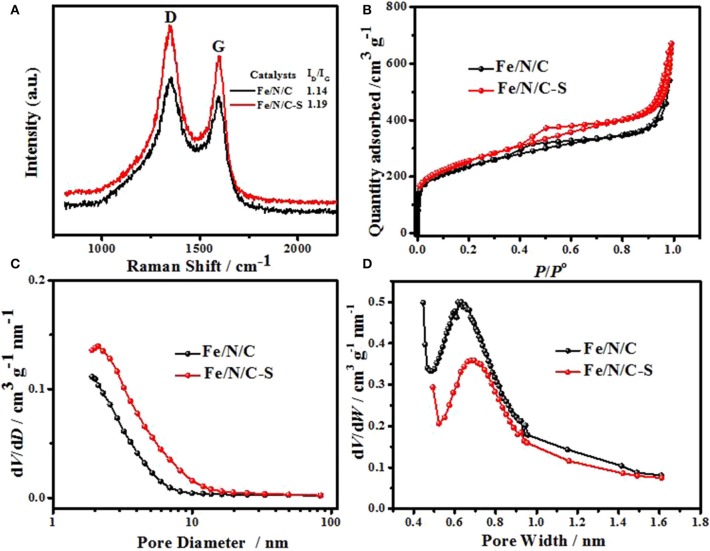
**(A)** Raman spectra, **(B)** Ar adsorption and desorption isotherms, **(C)** mesopore size distribution, and **(D)** micropore size distribution of Fe/N/C-S and Fe/N/C.

The graphitic carbon content in catalysts was tested via Raman spectroscopy. The characteristic D- (1350 cm^−1^) and G-band (1594 cm^−1^) confirmed the presence of graphitic carbon atoms structure in catalysts (Figure 4A), which is formed due to pyrolysis at high temperature. The graphitization and the defect density of carbon nanomaterials can be determined from peak intensities ratio of D- and G-band (Yang et al., 2019). While the intensity ratios *I*_D_/*I*_G_ values were calculated as 1.19 and 1.14 for Fe/N/C-S and Fe/N/C, respectively. Raman spectra analysis reveals that the Fe/N/C-S electrocatalyst has more defects as compared to the Fe/N/C, resulting in more electrocatalytic active sites. The porous structure of electrocatalysts was examined by Ar adsorption-desorption isotherms (Figure 4B). It is observed from the literature that the ORR activity also correlates with the Brunauer-Emmett-Teller (BET) surface areas of the electrocatalysts (Yang et al., 2018). The BET surface areas of Fe/N/C and Fe/N/C-S electrocatalysts are 738 and 830 m^2^g^−1^, respectively. It is noticed that surface areas of Fe/N/C-S electrocatalyst is significantly improved due to the heteroatoms doping (Fe, S, and N) as compared to the Fe/N/C electrocatalyst. With heteroatoms doping, the Ar adsorption-desorption curves displayed a typical isotherm type-IV and a capillary condensation phenomenon results from a relative pressure value of 0.3–0.8, which indicates the ordered mesoporous nanostructure. As comparison, S-doped electrocatalyst showed more mesoporosity and mesopore volume than Fe/N/C catalyst but less microporosity, as shown in Figures 4C,D. The doping of heteroatoms significantly improved the mesoporosity. Fe plays an especially imperative function in the formation of a mesoporous structure, increases the value of BET-specific surface areas and the mesopore volume of electrocatalysts. These structural features can accelerate the ORR process.

**Figure 5:**
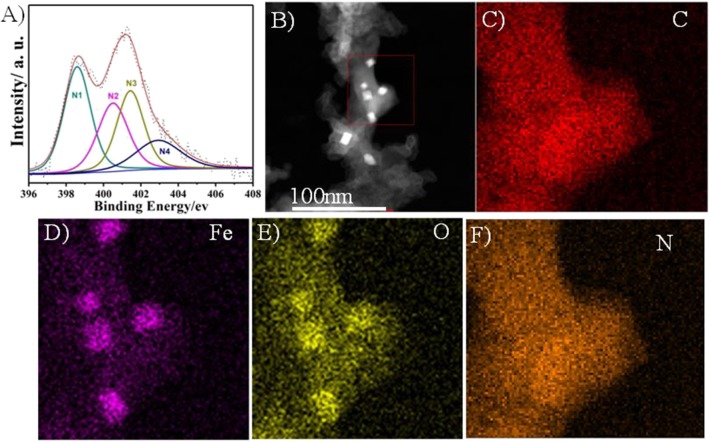
**(A)** The high-resolution N 1s spectrum, **(B)** ADF-STEM image, and **(C–F)** EDS elemental mapping of C, Fe, O and N, of Fe/N/C-S.

XPS and EDS were performed to confirm the heteroatoms doping in the prepared Fe/N/C-S electrocatalyst. Figure S1 shows the high-resolution S 2p spectra with three main peaks. The former two peaks correlated to S 2p3/2 and S 2p1/2 at binding energy values of 163.5 and 164.8 eV, respectively, which confirms the thiophene-like structure (C-S-C) in the carbon framework. The third peak at 168.6 eV was associated with the oxidized sulfur groups (C–SOx–C) at the surface of electrocatalyst. The heteroatoms (S, N, and Fe) can change the electronic structure and create new active centers in the electrocatalyst, which benefits the ORR process. The high-resolution N1s spectra of Fe/NC-S presents in Figure 5A. The N1s spectra of S and N-doped electrocatalyst were categorized into four different nitrogen peaks to get more insight into the surface composition and relative percentage of active N species. The different N-peaks are corresponding to N1 pyridinic (398.3 eV), N2 pyrrolic (400 eV), N3 graphitic (401 eV), and N4 oxidized pyridine (403.5eV), respectively. The relative percentage content of the N1, N2, N3, and N4 in the Fe/NC-S electrocatalyst were determined to be 27.3%, 22.7%, 34.6%, and 15.4 wt%, respectively. Both the pyridinic N1 and graphitic N3 may take part in the ORR process in alkaline solution (Wen et al., 2012). Both these N active species' (N1 and N3) percentage is 61.8% of the total nitrogen content in Fe/NC-S. The ORR performance of Fe/N/C-based electrocatalysts in acidic electrolyte is highly dependent on the percentage of pyridinic N. According to XPS analysis, the total weight contents of N, S, and Fe were calculated to be 3, 0.24, and 1.6% for the Fe/N/C electrocatalyst, as well as 6.3, 2.5, and 3.2% for the Fe/N/C-S catalyst, respectively. It can be seen that Fe and N content significantly increased in the Fe/N/C-S electrocatalyst in comparison with the Fe/N/C catalyst. Obviously, the S-doping can make possible the maintenance of Fe and N contents in S-doped electrocatalyst. Furthermore, annular dark-field scanning transmission electron microscopy (ADF-STEM) and EDS mapping analysis performed to see the elemental distribution in the best catalyst Fe/NC-S. The ADF-STEM and EDS mapping of C, Fe, O, and N were presented in Figures 5B–F. The elements have been uniformly distributed in the carbon framework, which may enhance the ORR performance in acidic as well as basic solution.

**Figure 6:**
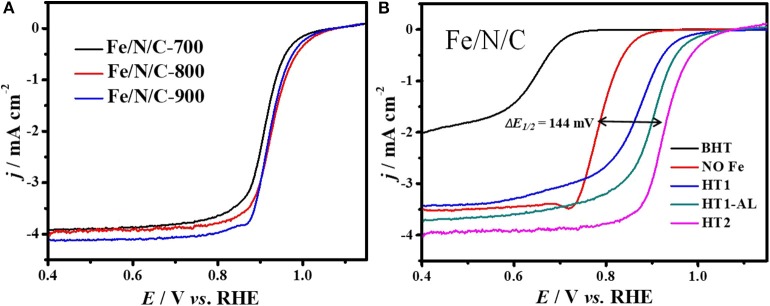
ORR polarization curves of Fe/N/C electrocatalysts **(A)** electrocatalysts synthesized at different temperatures, **(B)** electrocatalysts at different synthesis stages in O_2_-saturated 0.1 M NaOH solution at a scan rate of 10 mV s^−1^, 900 rpm.

The electrocatalytic performance of the Fe/N/C electrocatalyst for oxygen reduction reaction was investigated by using a cyclic voltammetry technique in the 0.1 M NaOH electrolyte. The electrolyte solution was saturated with O_2_ and N_2_ for 30 min prior to electrochemical tests. For comparison, a commercial Pt/C (20 wt% Pt) electrocatalyst was examined under similar conditions. For optimizing the synthetic conditions and ORR performance, we firstly determined the ORR activity of the electrocatalysts as a function of the pyrolysis temperature in the range of 700–900°C. The pyrolysis at high temperature played an important role in boosting the ORR activity for non-precious metal-based electrocatalysts (Wu et al., 2011b). The best ORR activity from the sample prepared at 800°C with high value of half-wave potential (944 mV) and onset-potential (110 mV) was observed in Figure 6A. At this high pyrolysis temperature, electrocatalysts may have high electron conductivity, active site density, and specific surface areas (Liu et al., 2010; Wu et al., 2011a; Ferrandon et al., 2012). It is reported that the relative percentage of nitrogen-active species in N-doped electrocatalysts significantly transformed between graphitic and pyridinic nitrogen due to heat treatment at high temperature (Li et al., 2009).

The function of cost-effective transition metals, such as Fe, Ni, Co, Mn, Cu, and Cr have been investigated as metal-ion centers for PGM-free electrocatalysts. Furthermore, the loading and content of these low-cost metals in NPM catalysts have also been subjected to investigation. The iron metal as dopant play a very significant role in enhancing the ORR performance in acidic and alkaline electrolyte solution, but the active sites in Fe-based electrocatalysts are still under debate. A number of studies have demonstrated that both Fe-metal and nitrogen doping have synergistic effects on ORR activities (Wen et al., 2012; Liu et al., 2013). It is noticed that electrocatalysts with and without Fe-content showed a big difference with respect to half-wave-potential value (Δ*E*_1/2_ = 144 mV). Figure 6B presents the ORR curves of Fe/N/C-catalyst at different synthesis steps (BHT-HT2). The capacitive current of polarization curves recorded in O_2_ and N_2_ has been subtracted to obtain the real current value for ORR (Figure S2). The shift in *E*_1/2_ potential value indicates that the oxygen reduction performance highly correlates with metal content in catalyst and FeN*x* moieties considered as active sites (Zagal et al., 2012; Tylus et al., 2014). Therefore, the Fe/N/C electrocatalyst showed best ORR performance with Fe-content at high pyrolyzing temperature 800°C, and 0.1 M H_2_SO_4_ acid solution used for acid leaching (Figure S3).

**Figure 7:**
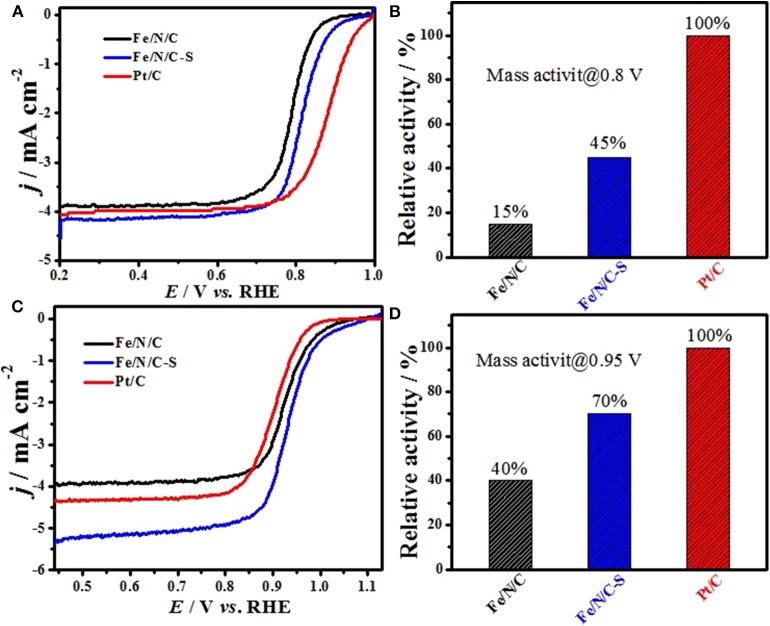
ORR polarization curves of Fe/N/C, Fe/N/C-S and Pt/C catalysts: **(A)** in 0.1 M H_2_SO_4_, **(B)** comparison of mass activity in alkaline media using Pt/C as reference, **(C)** polarization curves in 0.1 M NaOH, and **(D)** comparison of mass activity in acidic media using Pt/C as reference.

The ORR activity of Fe/N/C and S-doped Fe/N/C-S electrocatalysts was examined in alkaline and acidic electrolytes by RRDE technique. Figure 7A shows ORR polarization curves of Fe/N/C-S, Fe/N/C, and Pt/C electrocatalysts in O_2_-saturated 0.1 M H_2_SO_4_ electrolyte, with 900 rpm at a scan rate of 10 mV s^–1^. Clearly, the ORR electrocatalytic performance of an S-doped catalyst (Fe/N/C-S) in acid electrolyte is significantly improved as compared to the Fe/N/C catalyst. In 0.1 M H_2_SO_4_ acid solution, Fe/N/C-S performed the best ORR activity with an *E*_1/2_ potential value ca. 820 mV (RHE), which is only 64 mV less than commercial Pt/C catalyst (884 mV vs. RHE). The *E*_1/2_ potential of Fe/N/C-S is positively shifted by 30 mV in the acidic electrolyte. The shift of *E*_1/2_ shows that the ORR performance in acidic medium is considerably improved due to heteroatoms doping. The low activity of the Fe/N/C electrocatalyst may be due to low density of Fe-N*x* and protonation of active sites in acid solution, such as pyridinic N (Jiang et al., 2016; Rauf et al., 2016). The remarkable catalytic activity of the heteroatoms doped Fe/N/C-S electrocatalyst arisen due to a mesoporous nanostructure, high surface areas and the number of potential active sites, as well heteroatoms, reduced the over-potential by changing the charge distribution of adjoining carbon atoms and created a new active sites center in the catalyst (Wang et al., 2015; Shen et al., 2017). Even S-doped catalyst had shown higher ORR performance in comparison with recently published reports on dual doped (transition metals and heteroatoms) electrocatalysts (in Tables S1, S2). Figure 7B displays the comparative histogram of ORR mass activity of Fe-doped electrocatalysts and Pt/C (as reference) in acidic electrolyte solution at 0.80 V. The ORR mass activity of Fe/N/C and Fe/N/C-S electrocatalysts reached 15% and 45% to that of the Pt/C electrocatalyst, respectively. In 0.1 M NaOH solution, Fe/N/C-S also showed a higher oxygen reduction activity with an onset-potential of 1.10 V (RHE) and an *E*_1/2_ potential of 0.944 V, exceeding the Pt/C catalyst performance (a half-wave potential 0.91 V) in Figure 7C. Figure 7D demonstrates the comparison histogram of the ORR mass activity of PGM-free electrocatalysts with Pt/C as a reference catalyst in basic electrolyte at 0.95 V. The mass activity of Fe/N/C and S-doped Fe/N/C-S electrocatalysts are 40 and 70%, respectively to that of the Pt/C catalyst. As a result, the Fe/N/C-S electrocatalyst has greater current density and overall better electrochemical performance due to doping of heteroatoms (S and N), and a high mesoporous structure with a large value of surface area.

We further investigated the ORR efficiency of the Fe/N/C-S electrocatalyst and its corresponding reaction mechanism by the hydrogen peroxide (H_2_O_2_) yield. The ORR selectivity information mostly obtained from the H_2_O_2_ percentage. The ORR process includes three possible routes: one is direct four electrons transfer mechanism with high efficiency, and the others two are indirect routes via H_2_O_2_ formation as intermediate with low efficiency (Jaouen, 2009). The Fe/N/C-S catalyst exhibits the lowest (≥2) H_2_O_2_ yield in 0.1 M NaOH and 0.1 M H_2_SO_4_ electrolytes, even less (≥1%) at a high potential value between 0.60 to 0.90 V in Figure S4. The *n*_e_ value shows that the ORR on the Fe/N/C-S electrocatalyst followed the 4e-transfer process over O_2_ molecule in both acidic as well as basic electrolytes, which is owing to the synergistic effects caused by doping of S, N, and Fe atoms.

**Figure 8:**
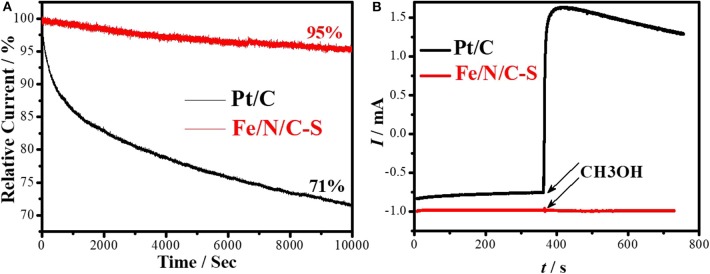
**(A)** Stability test of Fe/N/C-S and Pt/C catalysts at constant potential 0.8 V for 10,000 s. **(B)** Methanol tolerance (0.5 M conce.), in O_2_-saturated 0.1 M NaOH with Scan rate: 10 mV s^−1^; Rotating rate: 900 rpm.

The durability and resistance to the alcohol crossover effect are important for effective utilization of PGM-free electrocatalysts in fuel cells applications. The durability of the Fe/N/C-S and Pt/C electrocatalysts were scrutinized at 0.8 V in O_2_ saturated 0.1 M NaOH solution. The Pt/C catalyst was degraded 29% within 3 h while the Fe/N/C-S electrocatalyst lost only 5% of its initial activity at the same time (Figure 8A). It is evident that the decaying rate of the Fe/N/C-S electrocatalyst is slower as compared to Pt/C under alkaline ORR conditions. The methanol tolerance experiment was accomplished at 0.8 V. For comparison, the Pt/C electrocatalyst was tested in similar conditions as shown in Figure 8B. There was no effect on the surface of the Fe/N/C-S electrocatalyst upon the injection of CH_3_OH into the electrolyte. But, the current of Pt/C dramatically changed after the addition of methanol due to the electrooxidation of methanol molecules, and the carbon monoxide poisoning effect on the Pt/C electrocatalyst. It is known that CO is formed as a reaction intermediate during the methanol oxidation process, which is firmly adsorbed on the Pt surface. However, the current value was not constant in the case of the Pt/C catalyst as compared to Fe/N/C-S. This result indicated that the Fe/N/C-S electrocatalyst is a promising candidate for ORR in alkaline fuel cell applications.

## Conclusions

In conclusion, we prepared heteroatoms doped Fe/N/C electrocatalysts through high-temperature pyrolysis. As prepared, the N, S and Fe-doped Fe/N/C catalyst had shown outstanding ORR performances in both alkaline and acidic electrolytes. The main objectives of this study—to improve the ORR activity in acidic electrolyte—were achieved through the doping of heteroatoms. The electrocatalysts have high BET surface area with a mesoporous structure. The best catalyst exhibited high durability, tolerance to methanol, low H_2_O_2_ percentage, and follows the 4e transfer process. The mass activity of the Fe/N/C-S catalyst reached 45% in acidic medium and 70% in alkaline medium to that of reference Pt/C. The enhanced performance of the Fe/N/C-S electrocatalyst is comparable to Pt/C due to high surface area and active species, such as thiophene-S, graphitic N, pyridinic N, and Fe-N*x*. The dual doping of transition metals (Fe, Co) and heteroatoms (S and N) in non-noble catalysts can promote electrochemical performance. The Fe/N/C-S electrocatalyst is a promising candidate to replace conventional noble metal-based electrocatalysts in alkaline and acidic solutions.

## Data Availability Statement

All datasets generated for this study are included in the article/Supplementary Material.

## Author Contributions

All authors listed have made a substantial, direct and intellectual contribution to the work, and approved it for publication.

### Conflict of Interest

The authors declare that the research was conducted in the absence of any commercial or financial relationships that could be construed as a potential conflict of interest.
