# Supplementary material for: Novel Heteroatom-Doped Fe/N/C Electrocatalysts With Superior Activities for Oxygen Reduction Reaction in Both Acid and Alkaline Solutions
Source: Front Chem. 2020 Feb 18;8:78. doi: 10.3389/fchem.2020.00078 (PMC7040484; doi:10.3389/fchem.2020.00078)
Supplement: Supplementary file 1 [file Table_1.DOCX]

Supplementary Material

**Supplementary Figure 1** (A) S2p XPS spectra of Fe/N/C-S

**Supplementary Figure 2** (A) Original polarization curves of Fe/N/C (800 ^o^C) catalyst (loading, 1.0 mg cm^-2^) recorded in 0.1 M NaOH solution saturated with O_2_ (Black-solid line) and N_2_ (Red-solid line). (B) Background-subtracted ORR polarization curve.

Figure S2 (A) shows the original polarization curves of Fe/N/C (800℃) catalyst recorded in 0.1 M NaOH solution saturated with O_2_ and N_2_. The catalyst showed large capacitive current in the polarization curve recorded in N_2_-saturated solution due to high specific surface area of the catalyst. The ORR polarization curves are overlapped after capacitive current subtraction in Figure S2 (B).

We studied the ORR activity of the mesoporous Fe/N/C composit catalysts. These catalysts were leached with different concentration of acids (e.g, H_2_SO_4_ = 0.05 M, 0.1 M and HCl = 0.5 M, 1 M) in Figure S3). The iron content was decresed when highly concenrated acid use in acid leaching step.


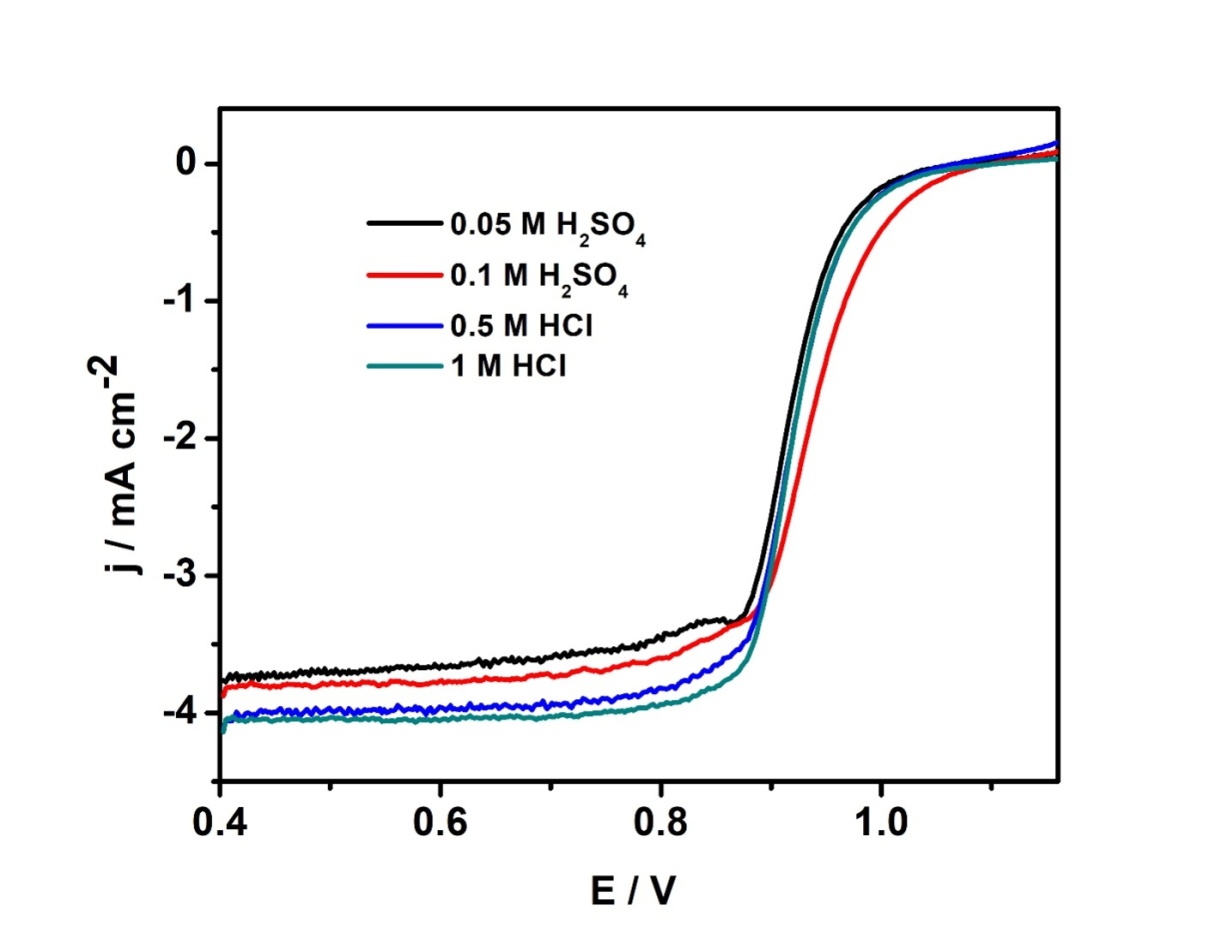


**Supplementary Figure 3.** ORR polarization curves of catalyst (loading, 1.0 mg cm^-2^) in O_2_-saturated 0.1 mol L^-1^ NaOH solution at a scan rate of 10 mV s^-1^, 900 rpm. Different concentrations of acids are used during acid leaching process.

**Supplementary Figure 4.** H_2_O_2_ yield and inset Electron- transfer number of Fe/NC-S catalyst in acidic and alkaline electrolyte.

**Supplementary Table 1** Comparison of ORR performance of Fe/N/C-S with other S-doped non-noble metal catalysts in alkaline medium.

| Electrocatalysts | *E_1/2_*(V  *vs.*RHE) | *E_onset_*(V *vs.*RHE) | Loading/mg cm^-2^ | Year Ref. |
| --- | --- | --- | --- | --- |
| Fe/N/C-S | **0.944** | **1.10** | **0.6** | **This work** |
| Fe/N/S-PC | 0.87 | 0.97 | 0.5 | 2018([Zheng et al., 2019](#_ENREF_15)) |
| FeS/Fe3C@N-S-C-800 | 0.87 | 1.02 | 0.3 | 2018([Kong et al., 2018](#_ENREF_8)) |
| Fe-SN-C@800 | 0.848 | 0.935 | 0.18 | 2018([Tong et al., 2018](#_ENREF_12)) |
| TETCB-Fe-N/S/C | 0.908 | 1.01 | 0.7 | 2018([Zhu et al., 2018](#_ENREF_16)) |
| Fe_14_NDC-9 | 0.888 | 0.968 | 0.6 | 2018([Hoque et al., 2018](#_ENREF_5)) |
| Fe–S/N–C-800 | 0.826 | 0.936 | 0.2 | 2017([Guo et al., 2017](#_ENREF_3)) |
| N, S-Fe/N/C-CNT | 0.85 | 0.98 | 0.6 | 2017([Chen et al., 2017](#_ENREF_2)) |
| Fe/SNC | 0.86 | 0.986 | 0.5 | 2017([Shen et al., 2017](#_ENREF_11)) |
| Fe-M-LA/C-700 | 0.78 | 0.93 | 0.2 | 2017([Huang et al., 2017](#_ENREF_7)) |
| S-Fe/N/C | 0.84 | 0.91 | 0.16 | 2016([Hu et al., 2016](#_ENREF_6)) |
| AT-Fe/N/C | 0.926 | 1.08 | 0.6 | 2015([Chen et al., 2015](#_ENREF_1)) |
| S/N_Fe27 | 0.87 | 0.93 | 0.8 | 2014([Ranjbar Sahraie et al., 2014](#_ENREF_9)) |

**Supplementary Table 2** Comparison of ORR performance of Fe/N/C-S with other S-doped non-noble metal catalysts in acidic medium.

| Electrocatalysts | *E_1/2_*(V  *Vs.*RHE) | *E_onset_*(V *vs.*RHE) | Loading/mg cm^-2^ | Year Ref. |
| --- | --- | --- | --- | --- |
| Fe/N/C-S | **0.82** | **0.989** | **0.6** | **This work** |
| Fe/N/S-PC | 0.785 | 0.890 | 0.5 | 2018([Zheng et al., 2019](#_ENREF_15)) |
| Fe-SN-C@800 | 0.640 | 0.747 | 0.18 | 2018([Tong et al., 2018](#_ENREF_12)) |
| TETCB-Fe-N/S/C | 0.79 | 0.899 | 0.7 | 2018([Zhu et al., 2018](#_ENREF_16)) |
| Fe_14_NDC-9-W_2M_-9 | 0.730 | 0.821 | 0.6 | 2018([Hoque et al., 2018](#_ENREF_5)) |
| S-Fe/N/C-16.4 | 0.820 | 0.975 | 0.6 | 2018([Wu et al., 2018](#_ENREF_14)) |
| Fe/SNC | 0.770 | 0.880 | 0.6 | 2017([Shen et al., 2017](#_ENREF_11)) |
| AT-Fe/N/C | 0.810 | 0.905 | 0.6 | 2016([Rauf et al., 2016](#_ENREF_10)) |
| S-Fe/N/C | 0.660 | 0.830 | 0.16 | 2016([Hu et al., 2016](#_ENREF_6)) |
| CoS_2_-CG | 0.660 | 0.780 | 0.6 | 2015([Higgins et al., 2015](#_ENREF_4)) |
| Fe/N/C-SNC | 0.836 | 0.985 | 0.6 | 2015([Wang et al., 2015](#_ENREF_13)) |

Herein, we compared the electrochemical performance of pyrolyzed heteroatoms based-electrocatalyst Fe/N/C-S (Table 1 and 2). For comparison, the published scientific papers were selected from recent five years. The electrode potentials of Hg/HgO electrode, Ag/AgCl electrode and SCE were converted into RHE scale. Following equations were used to convert the potential into RHE scale.

E(RHE) = E(Hg/HgO) + 0.918 V (5)

E(RHE) = E(Ag/AgCl) + 0.0591× pH + 0.197 V (6)

E(RHE) = E(SCE) + 1.002 V (7)

The possible error ranges of the potential values are ±0.02 V for all the onset potentials and the half-wave potentials, respectively.

**Reference:**

Chen, C., Yang, X.-D., Zhou, Z.-Y., Lai, Y.-J., Rauf, M., Wang, Y., Pan, J., Zhuang, L., Wang, Q., Wang, Y.-C., Tian, N., Zhang, X.-S., and Sun, S.-G. (2015). Aminothiazole-derived N,S,Fe-doped graphene nanosheets as high performance electrocatalysts for oxygen reduction. *Chemical Communications* 51**,** 17092-17095.

Chen, P., Zhou, T., Xing, L., Xu, K., Tong, Y., Xie, H., Zhang, L., Yan, W., Chu, W., Wu, C., and Xie, Y. (2017). Atomically Dispersed Iron–Nitrogen Species as Electrocatalysts for Bifunctional Oxygen Evolution and Reduction Reactions. *Angewandte Chemie International Edition* 56**,** 610-614.

Guo, J., Niu, Q., Yuan, Y., Maitlo, I., Nie, J., and Ma, G. (2017). Electrospun Core-Shell Nanofibers Derived Fe-S/N Depoed Carbon Material for Oxygen Reduction Reaction. *Applied Surface Science* 416**,** 118-123.

Higgins, D.C., Hassan, F.M., Seo, M.H., Choi, J.Y., Hoque, M.A., Lee, D.U., and Chen, Z. (2015). Shape-controlled octahedral cobalt disulfide nanoparticles supported on nitrogen and sulfur-doped graphene/carbon nanotube composites for oxygen reduction in acidic electrolyte. *Journal of Materials Chemistry A* 3**,** 6340-6350.

Hoque, M., Zhang, S., Thomas, M.L., Li, Z., Suzuki, S., Ando, A., Yanagi, M., Kobayashi, Y., Dokko, K., and Watanabe, M. (2018). Simple combination of a protic salt and an iron halide: precursor for a Fe, N and S co-doped catalyst for the oxygen reduction reaction in alkaline and acidic media. *Journal of Materials Chemistry A* 6**,** 1138-1149.

Hu, K., Tao, L., Liu, D., Huo, J., and Wang, S. (2016). Sulfur-Doped Fe/N/C Nanosheets as Highly Efficient Electrocatalysts for Oxygen Reduction Reaction. *ACS Applied Materials & Interfaces* 8**,** 19379-19385.

Huang, H.-C., Lin, Y.-C., Chang, S.-T., Liu, C.-C., Wang, K.-C., Jhong, H.-P., Lee, J.-F., and Wang, C.-H. (2017). Effect of a sulfur and nitrogen dual-doped Fe–N–S electrocatalyst for the oxygen reduction reaction. *Journal of Materials Chemistry A* 5**,** 19790-19799.

Kong, F., Fan, X., Kong, A., Zhou, Z., Zhang, X., and Shan, Y. (2018). Covalent Phenanthroline Framework Derived FeS@Fe3C Composite Nanoparticles Embedding in N-S-Codoped Carbons as Highly Efficient Trifunctional Electrocatalysts. *Advanced Functional Materials* 28**,** 1803973.

Ranjbar Sahraie, N., Paraknowitsch, J.P., Göbel, C., Thomas, A., and Strasser, P. (2014). Noble-Metal-Free Electrocatalysts with Enhanced ORR Performance by Task-Specific Functionalization of Carbon using Ionic Liquid Precursor Systems. *Journal of the American Chemical Society* 136**,** 14486-14497.

Rauf, M., Zhao, Y.-D., Wang, Y.-C., Zheng, Y.-P., Chen, C., Yang, X.-D., Zhou, Z.-Y., and Sun, S.-G. (2016). Insight into the different ORR catalytic activity of Fe/N/C between acidic and alkaline media: Protonation of pyridinic nitrogen. *Electrochemistry Communications* 73**,** 71-74.

Shen, H., Gracia-Espino, E., Ma, J., Zang, K., Luo, J., Wang, L., Gao, S., Mamat, X., Hu, G., Wagberg, T., and Guo, S. (2017). Synergistic Effects between Atomically Dispersed Fe−N−C and C−S−C for the Oxygen Reduction Reaction in Acidic Media. *Angewandte Chemie International Edition* 56**,** 13800-13804.

Tong, J., Li, W., Ma, J., Wang, W., Bo, L., Lei, Z., and Mahboob, A. (2018). Nitrogen and Sulfur Dual Self-Doped Graphitic Carbon with Highly Catalytic Activity for Oxygen Reduction Reaction. *ACS Applied Energy Materials*.

Wang, Y.-C., Lai, Y.-J., Song, L., Zhou, Z.-Y., Liu, J.-G., Wang, Q., Yang, X.-D., Chen, C., Shi, W., Zheng, Y.-P., Rauf, M., and Sun, S.-G. (2015). S-Doping of an Fe/N/C ORR Catalyst for Polymer Electrolyte Membrane Fuel Cells with High Power Density. *Angewandte Chemie International Edition* 54**,** 9907-9910.

Wu, Y.-J., Wang, Y.-C., Wang, R.-X., Zhang, P.-F., Yang, X.-D., Yang, H.-J., Li, J.-T., Zhou, Y., Zhou, Z.-Y., and Sun, S.-G. (2018). Three-Dimensional Networks of S-Doped Fe/N/C with Hierarchical Porosity for Efficient Oxygen Reduction in Polymer Electrolyte Membrane Fuel Cells. *ACS Applied Materials & Interfaces* 10**,** 14602-14613.

Zheng, L., Dong, Y., Chi, B., Cui, Z., Deng, Y., Shi, X., Du, L., and Liao, S. (2019). UIO-66-NH2 -Derived Mesoporous Carbon Catalyst Co-Doped with Fe/N/S as Highly Efficient Cathode Catalyst for PEMFCs. *Small* 15**,** e1803520.

Zhu, Y., Chen, X., Liu, J., Zhang, J., Xu, D., Peng, W., Li, Y., Zhang, G., Zhang, F., and Fan, X. (2018). Rational Design of Fe/N/S-Doped Nanoporous Carbon Catalysts from Covalent Triazine Frameworks for Efficient Oxygen Reduction. *ChemSusChem* 11**,** 2402-2409.
